# Seminal Fluid Protein Acp29AB Shifts Egg‐Laying Timing in *Drosophila* Without Detectable Effects on Female Fitness

**DOI:** 10.1002/ece3.72677

**Published:** 2025-12-12

**Authors:** Samantha Kincaid, Graham C. McLaughlin, Sophia Sudak, Brian Hollis

**Affiliations:** ^1^ Department of Biological Sciences University of South Carolina Columbia South Carolina USA

**Keywords:** Acp29AB, *Drosophila melanogaster*, oviposition, RNAi, seminal proteins, sexual conflict

## Abstract

Seminal fluid proteins (SFPs) can alter female physiology and behavior, with consequences that may align with or oppose female interests. Many SFPs are known to shape aspects of the female post‐mating response, yet evidence for direct effects on female fitness is scarce. In *Drosophila melanogaster* , the SFP Acp29AB is firmly linked to sperm defense (P1) and sperm storage, but its impacts on females are poorly understood. Using a GAL4–UAS RNAi system with perfectly matched genetic backgrounds, we manipulated *Acp29AB* gene expression either ubiquitously or specifically in the male accessory gland and then asked how knockdown alters male sperm competition and, in female mates, oviposition dynamics and competitive fitness. Both knockdowns impaired sperm defense, consistent with past work on Acp29AB. We uncovered a previously undetected effect on oviposition timing: across 14 days after a single mating, females mated to control males laid more eggs early in life than females mated to knockdown males, advancing the timing of reproduction. With accessory gland‐specific knockdown, this shift occurred without a change in total egg output, which may explain why earlier work focused on totals alone did not detect an effect. Despite accelerated oviposition in mates of control males, female competitive fitness in a five‐day, early‐life assay that included direct (female–female) and indirect (larval) competition did not differ between treatments. Thus, Acp29AB enhances male sperm defense and promotes earlier egg laying in mates, but under our experimental conditions has no detectable net effect on female fitness. These results broaden the functional scope of Acp29AB to include female reproductive schedule and suggest that some SFPs may evolve rapidly while either being effectively neutral or having context‐dependent effects on female fitness.

## Graphical abstract

Seminal fluid proteins (SFPs) can profoundly affect female reproduction, but direct fitness consequences are often unclear. We show that the *Drosophila* SFP Acp29AB contributes to male sperm defense and advances the timing of female egg laying without altering total egg output or competitive female fitness. These results broaden Acp29AB's functional role to include reproductive scheduling and highlight how SFPs can shape post‐mating dynamics with context‐dependent fitness effects on females.

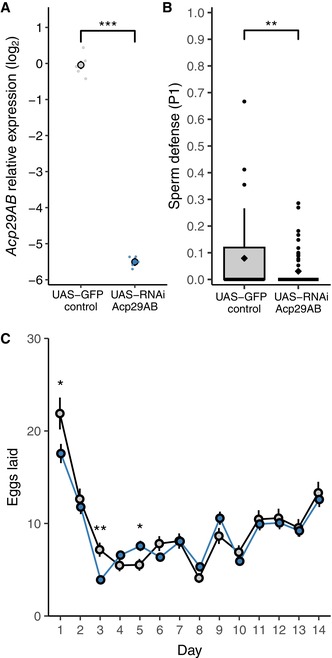

## 1 Introduction

Males and females share an evolutionary goal of successful reproduction, necessitating some degree of cooperation between the sexes. The strategies that maximize fitness for each sex often diverge, however, which generates sexual conflict (Parker 1979; Arnqvist and Rowe 2005). One way this sexual conflict might manifest is through the transfer of seminal fluid proteins (SFPs) (Sirot et al. 2015). In many animals, males transfer SFPs to females during mating, where they alter female physiology and behavior in ways that can benefit one sex while imposing costs on the other. Among insects, the fruit fly *Drosophila melanogaster* is the best‐characterized system, where SFPs elicit a suite of responses known as the female post‐mating response (PMR) that shapes reproductive outcomes in ways that can align with or oppose female interests (Chapman 2001; Sirot et al. 2015; Wolfner 2002, 2009).

Evolutionary patterns from comparative work and experimental evolution provide some insight into the selection pressures on SFPs as a group. Comparative studies show that SFP genes in *Drosophila* evolve rapidly, with elevated ratios of non‐synonymous to synonymous substitutions that typically signal strong positive selection (Haerty et al. 2007; Mueller et al. 2005; Swanson et al. 2001). Yet this apparent signature of adaptive change is accompanied by high levels of genetic polymorphism and an excess of rare alleles (Patlar et al. 2021), patterns more consistent with relaxed selection than with coevolutionary arms races between the sexes. This raises the possibility that at least some SFPs may actually be evolving rapidly because they are neutral in their effects on male and female fitness. By contrast, experimental evolution after the removal of sexual conflict has repeatedly led to males that harm females less (e.g., Crudgington et al. 2010; Holland and Rice 1999; Hollis et al. 2019). Although such changes need not be mediated by SFPs, in at least one case males also evolved a coordinated downregulation of SFP gene expression once conflict was removed (Hollis et al. 2019), implying that SFPs as a whole impose costs on females. Those same males induced less early egg laying in their mates, however, suggesting that receipt of normal levels of SFPs may benefit females in contexts where early‐life reproduction contributes more to fitness than later reproduction. Further, the coordinated downregulation of SFP expression under reduced conflict could reflect shared regulatory control of the group, masking any heterogeneity in the costs and benefits to females of individual proteins.

The most well‐studied SFP in *D. melanogaster* is sex peptide (SP), notable for effects on remating, egg production, lifespan, activity, and endocrine and immune function, but the balance of its effects on female fitness is still uncertain (Hopkins and Perry 2022). SP exemplifies the uncertainty over whether SFP effects are primarily antagonistic or cooperative. On one hand, SP shortens female lifespan, an apparent cost (Wigby and Chapman 2005). On the other hand, these effects on lifespan do not appear until laboratory females are several weeks old, a window that may not reflect biologically realistic patterns of egg production in nature and could therefore represent little to no fitness cost. Moreover, even if there are costs from earlier death, these may be offset by the well‐established early effects of SP on egg production, which are expected to yield fitness benefits under ecological conditions where early reproduction is favored. Consistent with this idea, lifetime reproductive success of females from at least some *D. melanogaster* genetic backgrounds is higher when females receive SP during mating than when they do not (Wensing and Fricke 2018). The combination of costs and benefits has made SP a focal point in the debate over conflict and cooperation, but its breadth of action simultaneously makes net effects on females difficult to resolve and limits generalizability to other SFPs.

Beyond SP, there are ~300 other SFPs in *D. melanogaster* (Wigby et al. 2020) and only a few have been functionally characterized. Most appear to have narrower, more specialized roles than SP, often targeting specific aspects of post‐mating reproductive biology. Among these, several SFPs have been found to play a critical role in sperm storage (Avila et al. 2015; Avila and Wolfner 2009; Neubaum and Wolfner 1999), a process that sits at the intersection of male and female interests because it underpins male paternity success and is essential for female fertility. A well‐characterized example of an SFP gene implicated in sperm storage is *Acp29AB*, which was originally found to be associated with competitive paternity share (Clark et al. 1995; Fiumera et al. 2005) and shows evidence of directional selection at both sequence (Aguadé 1999) and expression (Flacchi et al. 2024) levels. Acp29AB is a lectin that localizes to female sperm storage organs after mating and promotes sperm storage (Wong et al. 2008). Acp29AB contributes to P1 (the proportion of progeny sired by the first male to mate) but not P2 (the proportion of progeny sired by the second male). These functions clearly shape male reproductive outcomes and might also be expected to reduce female fitness based on past work that found a correlation between male sperm competition success and the mortality of mates (Civetta and Clark 2000). The effects of receipt of Acp29AB on female fitness remain unclear, however. Wong et al. (2008) found no differences when looking at total egg output or total offspring number when females received Acp29AB versus when they did not, suggesting its effects may be largely male‐limited. Similarly, a recent study by Patlar and Civetta (2022) reported no differences in male fertility that depended on *Acp29AB* expression levels, but did not track female oviposition. Neither study addressed the timing of egg laying or the competitive contexts that may shape female fitness. Thus, despite established roles in male reproductive success, the effects of Acp29AB on females remain incompletely understood. Indeed, the same measured quantity (counts of emerged offspring) was interpreted as female fertility in one study (Wong et al. 2008) and as male fertility in the other (Patlar and Civetta 2022), highlighting the extent to which post‐mating phenotypes arise from joint male–female interactions and resist simple assignment to one sex or the other.

Here, we set out to assess more directly the impact of Acp29AB on female reproduction and fitness. We used a GAL4–UAS RNAi system with both a ubiquitous and a tissue‐specific driver to knock down *Acp29AB* expression. Importantly, all experimental flies were perfectly matched for genetic background outside of the UAS‐RNAi element, a level of genetic control that was not obtained in previous RNAi studies. With this design in place, we pursued three aims. First, we confirmed the established sperm competition phenotype (sperm defense or P1) as a benchmark for validating our manipulation. Second, we examined whether Acp29AB affects the timing and total output of female egg laying, tracked with daily resolution over a two‐week period following mating. Finally, we asked whether detected effects in females translate into net fitness costs or benefits, using an assay design that emphasizes early‐life reproduction and includes both competition between females and competition between developing offspring. Together, this framework allowed us to examine both male benefits and female outcomes, and to evaluate whether the effects of Acp29AB are most consistent with male–female conflict, cooperation, or neutrality.

## 2 Materials and Methods

### 2.1 Fly Stocks and Rearing

Two different GAL4 drivers were used in the experiments. First, a ubiquitously expressed driver, *ubi*‐GAL4 (BDSC 32551), was used to drive expression throughout the fly. Because ubiquitous GAL4 expression can have negative effects on flies (Liu and Lehmann 2008), we also used a tissue‐specific driver, *ovu*‐GAL4 (courtesy M.F. Wolfner), that drives expression only in the male accessory glands. The UAS‐RNAi line for *Acp29AB* was created as part of the Transgenic RNAi Project (TRiP) using targeted phiC31 integration (Perkins et al. 2015) with an attP40 landing site, obtained from the Bloomington Drosophila Stock Center (BDSC 77372). The corresponding UAS‐GFP control line was also generated by TRiP using the same approach, with an attP2 landing site (BDSC 35786). Experimental flies were generated by crossing GAL4 driver line females to either UAS‐RNAi or UAS‐GFP males. Because the UAS‐GFP and UAS‐RNAi lines were generated in the same genetic background (Perkins et al. 2015), experimental flies are genetically matched outside of the UAS element. Experimental males expressing either the RNAi element, knocking down expression of *Acp29AB*, or the GFP element, our control with normal expression levels of *Acp29AB*, were collected as virgins from this cross.

Experiments with male or female competitors used flies carrying an *ebony* mutation as a competitive standard. These flies were obtained from the IVe population, which was established in 1992 from a spontaneous recessive *ebony* mutant in the Ives (IV) population (Houle and Rowe 2003). Homozygous *ebony* flies can be easily distinguished from wild‐type flies by body coloration, which enabled determination of competitive paternity success. Offspring from *ebony* females that were sired by knockdown or control males in sperm competition experiments were wild type in coloration, while offspring sired by *ebony* males were dark in coloration. To maintain consistency throughout our experiments, *ebony* females were also used in all other assays. Experimental flies were collected as virgins and used in experiments between 2‐ and 3‐days post‐emergence, matched between treatments within experimental blocks.

### 2.2 Confirming Gene Expression Knockdown by RNAi

We assessed knockdown efficiency using five biological replicates each of knockdown and control males. Five virgin males were collected and sacrificed at 3 days of age for each biological replicate, after which whole bodies were homogenized and RNA was extracted using the RNeasy Mini Kit (Qiagen). RNA concentration and quality were evaluated with a spectrophotometer and a Bioanalyzer (Agilent). Samples were treated with DNase (ArcticZymes gDNA removal kit) prior to cDNA synthesis with qScript XLT cDNA SuperMix (Quantabio). Quantitative PCR was performed using SYBR Green FastMix (Quantabio) in 10 μL reactions, with three technical replicates per biological replicate. Four housekeeping genes (*Act42A*, *αTub84B*, *Ef1α48D*, and *RpL13A*) were used as references; primer sequences and efficiencies are reported in Hollis et al. (2019). In preliminary experiments, we found no off‐target effects of UAS‐RNAi Acp29AB on the relative expression levels of four other tested SFPs (*Acp26Aa*, *Acp36DE*, *Acp62F*, and *SP*). Relative expression was quantified using an efficiency‐corrected ΔΔCt approach (Pfaffl 2001), incorporating all four housekeeping genes as references.

### 2.3 Sperm Defense (P1)

To measure sperm defense (P1), *ebony* females were placed individually in vials with either control or knockdown males and observed for 3 h. Males were then removed from vials in which copulation was observed, after which mated females were maintained in isolation for 2 days. To allow remating, four *ebony* males were then added to each vial for 1 day. Females were subsequently transferred to new vials and allowed to lay eggs for 2 days, after which they were discarded. Offspring were counted and scored for *ebony* versus wild‐type body coloration. P1 was calculated as the fraction of progeny that were wild type, that is, sired by the first male to mate. Females producing fewer than five progeny or failing to remate during the assay (no *ebony* progeny) were excluded from analyses.

### 2.4 Sperm Offense (P2)

Although previous work has not linked Acp29AB to sperm offense (P2), we measured P2 for the tissue‐specific driver to determine whether the P1 effects we observed extended to second‐male success. P2 was measured in a similar manner to P1, but with the order of males reversed. *Ebony* females were first placed individually in vials with *ebony* males and observed for 3 h. Males were removed from vials in which copulation was observed, and mated females were maintained in isolation for 2 days. To allow a second mating, one control or knockdown male was then added to each vial for 1 day. Females were subsequently transferred to new vials and allowed to lay eggs for 2 days, after which they were discarded. Offspring were counted and scored for *ebony* versus wild‐type body coloration. P2 was calculated as the fraction of progeny that were wild type, that is, sired by the second male to mate. Females producing fewer than five progeny or failing to remate (no wild‐type progeny) were excluded from analyses.

### 2.5 Egg‐Laying Time Series

Egg laying was measured in females mated to either control or knockdown males across a 14‐day period. Females were first individually paired with control or knockdown males and observed for 3 h. Females from successful matings were retained, and male partners were removed from vials. Each day for 14 days, females were transferred to a new vial and eggs laid during the preceding 24 h were counted. To facilitate egg counting, food coloring was added to the medium to darken the substrate and improve contrast with eggs. Females that laid fewer than ten eggs in total during the first 2 days were excluded from analyses.

### 2.6 Female Competitive Fitness

We measured the competitive fitness of females mated to either control or knockdown males using the *ovu‐*GAL4 driver in a design emphasizing early‐life reproduction and competition between females both directly (female–female interactions) and indirectly (larval competition). Ebony females were paired with either ebony males, control males, or knockdown males and observed for 3 h. Females that mated to control or knockdown males during this period were then paired with females that had mated to ebony males, and each pair was transferred to a new vial. Female pairs were allowed to lay eggs for 5 days, after which females were discarded. Progeny were counted and scored for ebony versus wild‐type body coloration. Competitive female fitness was calculated as the fraction of progeny that were wild‐type and compared between females mated to control versus knockdown males.

### 2.7 Statistical Analyses

All analyses were performed in R v4.3.1 (R Core Team 2023). Relative expression of *Acp29AB* (log_2_) was analyzed with a linear model including treatment (control vs. knockdown) as a fixed effect, and significance was assessed with likelihood ratio tests comparing the full model to a reduced model without treatment. Sperm defense (P1) and sperm offense (P2) values were non‐normal and zero/one‐inflated, so they were analyzed using Mann–Whitney *U* tests. For the *ovu*‐GAL4 driver, P1 data were collected in two experimental blocks. We therefore analyzed blocks separately and combined *p*‐values with Fisher's method, then performed a pooled analysis including both blocks. Results were similar across approaches. A Kolmogorov–Smirnov test did not detect differences in P1 distributions between blocks, so data were pooled for visualization. Egg‐laying data were analyzed with generalized linear mixed models in package *lme4* (Bates et al. 2015) with a Poisson error distribution and log link function. The number of eggs was modeled with treatment (control vs. knockdown mate identity), day, and the treatment × day interaction as fixed effects, and a random intercept of female to account for repeated measures. Significance was tested with likelihood ratio tests in package *afex* (Singmann et al. 2024), and treatment contrasts at individual days were evaluated with the pairs function in package *emmeans* (Lenth 2024). Female competitive fitness was analyzed with a generalized linear mixed model with a binomial error distribution and logit link function. Offspring identity (wild type vs. ebony) was modeled with treatment (control vs. knockdown mate identity) as a fixed effect and random effects of experimental block and female.

## 3 Results

### 3.1 Ubiquitous RNAi Knockdown of Acp29AB and Sperm Competition

**FIGURE 1:**
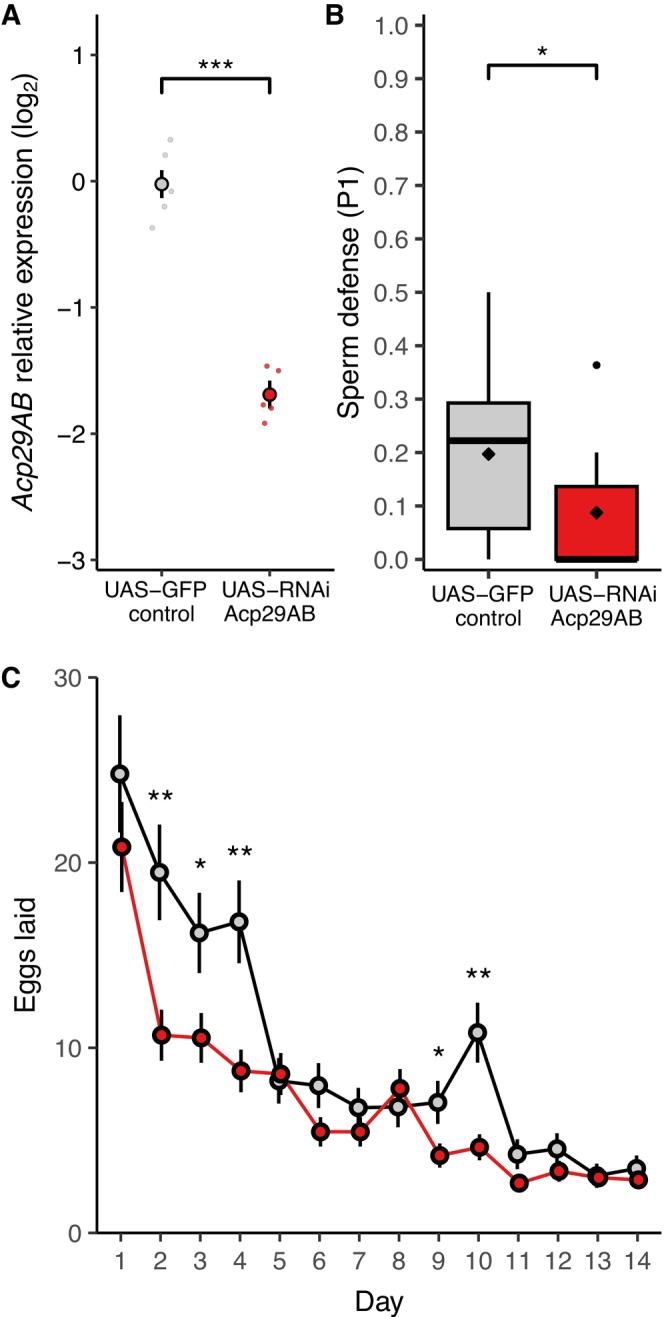
Whole‐fly knockdown of *Acp29AB* with RNAi (*ubi*‐GAL4 driver). (A) Relative expression (log_2_) of control and knockdown males (*n* = 5 biological replicates per group, each consisting of 5 males). (B) Measures of sperm defense (P1) in control and knockdown males (*n* = 9–15 per group), diamonds indicate mean. (C) Egg‐laying for females mated to either control or knockdown males across the first 14 days after mating (*n* = 14–16 per group/day). UAS‐GFP control males are depicted in gray, *Acp29AB* knockdown males are in red. Asterisks indicate significant differences between control and knockdown‐mated females in egg production for individual days. *p < 0.05, **p < 0.01, ***p < 0.001.

We first assessed Acp29AB knockdown efficiency and measured male sperm defense, a phenotype linked to Acp29AB in prior studies and therefore a key benchmark. We used a ubiquitously expressed GAL4 driver, *ubi*‐GAL4, to drive a UAS‐RNAi knockdown of *Acp29AB*. Gene expression was reduced in knockdown males to 31% that of control males (*χ* ^2^(1) = 27.25, *p* < 0.001, Figure 1A). With knockdown confirmed, we examined whether reduced *Acp29AB* expression impaired sperm defense (P1). As expected for P1 under last‐male sperm precedence, measures of P1 were low even for controls (median 22%), but much lower in knockdown males where the majority of males failed to sire any offspring at all (median 0%, Mann–Whitney *U* test, *U* = 96, *p* = 0.043, Figure 1B).

### 3.2 Ubiquitous Acp29AB Knockdown Males Reduce Female Egg Laying

We next measured the effects of control and knockdown males on female oviposition across the 3 weeks after mating (Figure 1C). Females laid more eggs in the days immediately after mating than later in the time course, with output declining steadily (day effect: *χ* ^2^(13) = 1182.03, *p* < 0.001). There was evidence that females mated to control males laid more eggs overall than those mated to knockdown males, although this effect was only marginally significant (treatment effect: *χ* ^2^(1) = 3.97, *p* = 0.046). Importantly, the difference in egg laying between females mated to control males and those mated to knockdown males depended strongly on day (treatment × day interaction: *χ* ^2^(13) = 60.08, *p* < 0.001): nearly all the reduction in egg laying by knockdown‐mated females occurred during the first 4 days after mating, after which egg laying rates largely converged between treatments outside of a brief window near the end of the 2‐week period.

### 3.3 Tissue‐Specific RNAi Knockdown of *Acp29AB* and Sperm Competition

**FIGURE 2:**
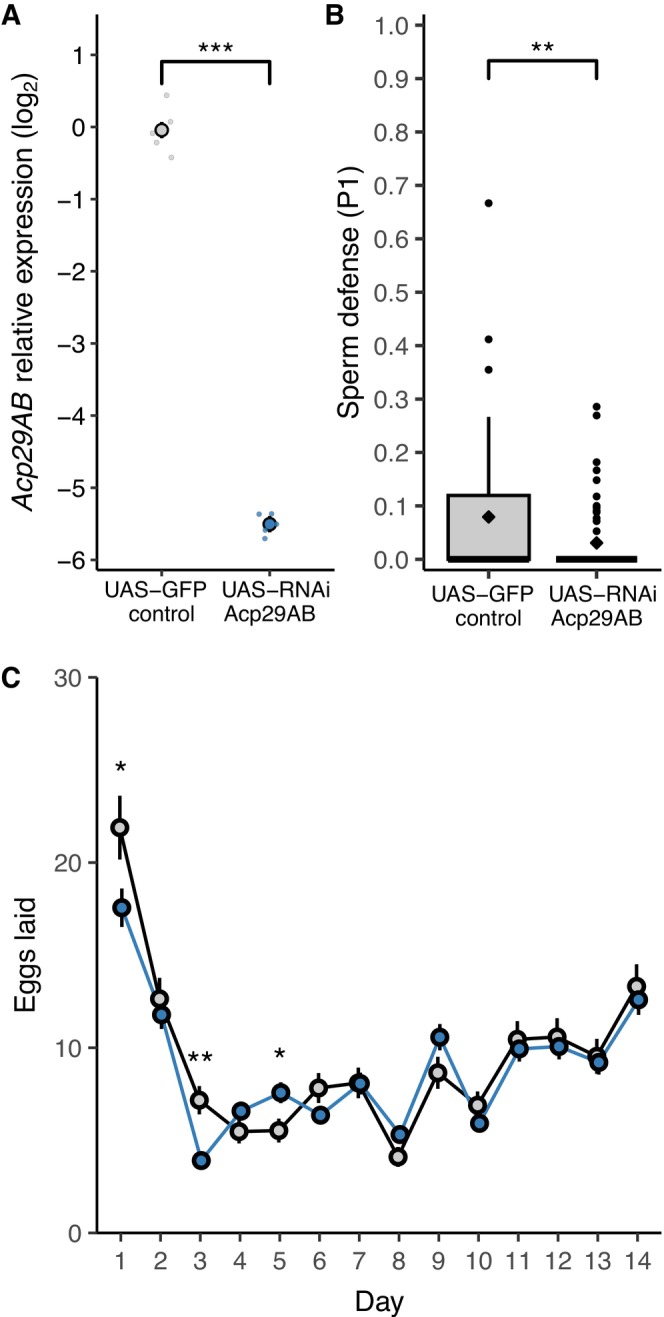
Tissue‐specific knockdown of *Acp29AB* with RNAi (*ovu*‐GAL4 driver). (A) Relative expression (log_2_) of control and knockdown males (*n* = 5 biological replicates per group, each consisting of 5 males). (B) Measures of sperm defense (P1) in control and knockdown males (*n* = 46–57 per group), diamonds indicate mean. (C) Egg‐laying for females mated to either control or knockdown males across the first 14 days after mating (*n* = 18–35 per group/day). UAS‐GFP control males are depicted in gray, *Acp29AB* knockdown males are in blue. Asterisks indicate significant differences between control and knockdown‐mated females in egg production for individual days. *p < 0.05, **p < 0.01, ***p < 0.001.

Our results with ubiquitous knockdown experiments suggested a role for Acp29AB in promoting early oviposition in females. It is possible, however, that this pattern could be driven by negative effects of expressing either GAL4 or RNAi throughout males. To examine this possibility, we used *ovu*‐GAL4 to drive RNAi specifically in the male accessory glands. Expression of *Acp29AB* was nearly eliminated in these knockdown males that exhibited approximately 2% of the levels of control males (*χ* ^2^(1) = 49.96, *p* < 0.001, Figure 2A). Targeted knockdown again reduced male sperm defense—P1 was low in both groups, but significantly lower in knockdowns (median 0%, mean 3%) than controls (median 0%, mean 8%, Mann–Whitney *U* test, *U* = 1615, *p* = 0.008, Figure 2B). With our tissue‐specific driver, we found that sperm offense (P2) did not differ significantly between control (median 100%) and knockdown males (median 96%, Mann–Whitney *U* test, *U* = 733, *p* = 0.138, Figure S1).

### 3.4 Tissue‐Specific Acp29AB Knockdown Males Reduce Early Egg Laying Without Affecting Total Output

We measured the effects of tissue‐specific knockdown on female oviposition over the 2 weeks following mating (Figure 2C). Female egg laying was higher in the days immediately after mating and diminished over time (day effect: *χ* ^2^(13) = 971.06, *p* < 0.001). Unlike the results from our ubiquitous GAL4 driver, however, tissue‐specific knockdown of *Acp29AB* did not result in reduced total oviposition of females mated to knockdown males (treatment effect: *χ* ^2^(1) = 0.20, *p* = 0.657). We again found strong evidence for a day‐dependent effect of knockdown (treatment × day interaction: *χ* ^2^(13) = 57.96, *p* < 0.001). In the first 3 days after mating, females mated to control males laid more eggs than those mated to knockdown males. After this period, knockdown‐mated females appeared to exhibit a short‐lived period of higher egg‐laying on days 4–5, but this pattern did not persist and egg‐laying rates subsequently equalized.

### 3.5 No Difference in Competitive Fitness for Females Mated to Acp29AB Knockdown Males

**FIGURE 3:**
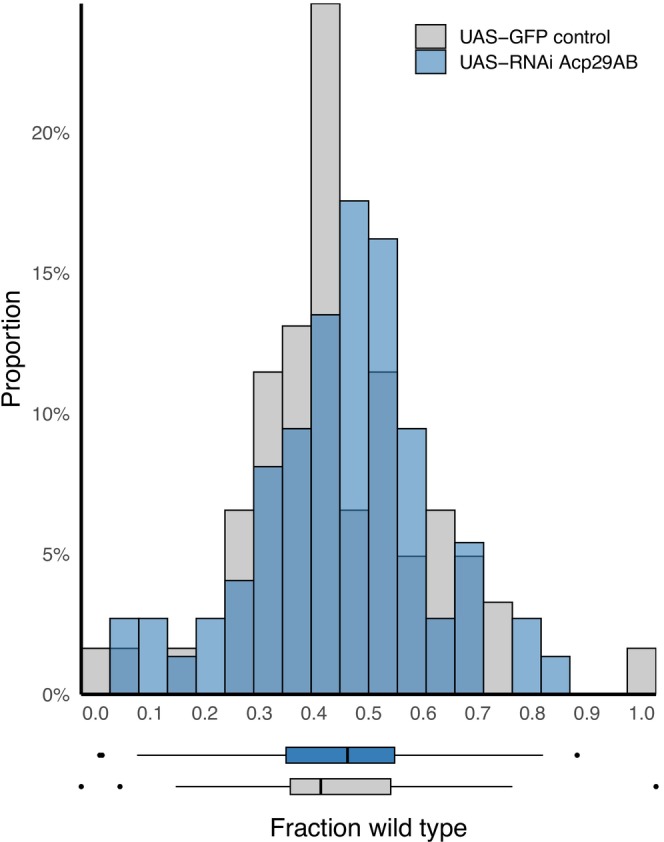
No difference in fitness for females mated to control versus knockdown (*ovu*‐GAL4 driver) males when placed in competition with *ebony*‐mated females. For each vial, the fraction of offspring that are wild‐type (and therefore came from control or knockdown‐mated females, as opposed to *ebony*‐mated female competitors) is shown (*n* = 64–71 per group). Females mated to control males are depicted in gray and those mated to *Acp29AB* knockdown males are in blue.

Based on our egg‐laying results showing higher egg‐laying in the days immediately following mating when females mated with control males, we reasoned that females might experience a net benefit from receipt of Acp29AB. We designed an assay to measure competitive fitness of females mated to either control or knockdown males that emphasized early‐life reproduction (5 days of egg‐laying) and captured competition with other females (e.g., for egg‐laying substrate) as well as any potential effects of SFP receipt on the downstream competitive ability of larvae. We used our tissue‐specific driver for these experiments, reasoning that the subtler effects detected on female egg‐laying with this driver were more biologically relevant. When females mated to control or knockdown males were placed in competition with *ebony* females in this assay, there was no difference in the fraction of total offspring that were produced by females mated to each type of male across three experimental blocks (Figure 3, treatment: *χ* ^2^(1) = 0.05, *p* = 0.830). Control‐mated female offspring made up on average 43.5% (95% confidence interval 38.9%–48.1%) of emerging offspring, while knockdown‐mated female offspring made up on average 44.1% (95% confidence interval 39.9%–48.4%).

## 4 Discussion

Our goal in this study was to assess how the seminal fluid protein Acp29AB influences female reproduction and fitness, alongside its established role in male sperm competition. We confirmed that Acp29AB knockdown impaired sperm defense, consistent with earlier work (Wong et al. 2008). Beyond this expected effect, however, we identified a novel role for Acp29AB in promoting increased oviposition by females in the days immediately following mating. Despite this change in reproductive timing, we found no evidence that the receipt of Acp29AB altered female competitive fitness.

In the only previous study that measured the impact of Acp29AB on female egg‐laying, no effects were detected (Wong et al. 2008). That study used an Acp29AB loss‐of‐function mutation and measured total oviposition across 10 days. We also do not see differences in total oviposition with our tissue‐specific manipulation of Acp29AB, indicating that egg‐laying should be assessed with time‐resolved series rather than totals alone. The time‐sensitive effect on earlier oviposition caused by Acp29AB parallels the effects of two of the most well‐characterized SFPs, Acp70A (sex peptide) and Acp26Aa (ovulin) (Chapman 2001; Sirot et al. 2015), and at least one other, CG33943 (Ram and Wolfner 2007). Our results suggest that SFPs causing temporal shifts in reproductive effort might be more widespread than appreciated, in particular for those SFPs like Acp70A, Acp26Aa, and Acp29AB, which have been recently identified as central, highly connected nodes in a rapidly evolving SFP subnetwork (Ranz et al. 2025). Shifting female reproductive effort toward the immediate post‐mating period could reflect a common strategy by which males front‐load their reproductive gains. Regardless, our findings broaden the scope of known Acp29AB function by showing its influence extends beyond sperm storage and fertilization outcomes to female reproductive scheduling.

The genetic manipulation in our study is unusually clean in that genetic backgrounds are matched aside from the UAS‐RNAi or UAS‐GFP elements. Because SFP studies often lack matched controls (e.g., entirely different genetic backgrounds in Patlar and Civetta (2022), whole chromosome differences in Ram and Wolfner (2007)), our matched design increases confidence that the observed effects reflect Acp29AB knockdown rather than background differences. That said, as with any RNAi approach, there are several other caveats. First, there is the possibility of off‐target effects of the RNAi construct. TRiP RNAi lines are designed (and have been shown experimentally) to minimize this risk (Perkins et al. 2015), and we further confirmed that our manipulation did not alter expression of four other SFPs, but off‐target effects cannot be excluded entirely. If off‐target effects were present, they would likely reduce overall reproduction rather than cause the specific timing shift we observed.

A second consideration concerns the driver used for knockdown. Ubiquitous knockdown reduced both early and total oviposition in our study, whereas accessory gland–specific knockdown affected only early oviposition. Because the ubiquitous driver achieved weaker knockdown in males but a stronger phenotype in female mates, the most parsimonious explanation for the observed difference between drivers is that widespread expression of GAL4 itself imposes costs (Liu and Lehmann 2008). This motivated our use of the accessory gland driver, which restricts both GAL4 and RNAi to the relevant tissue. Under this design, with nearly complete Acp29AB knockdown, both male P1 and female oviposition timing effects remained but were more modest and detectable only on a subset of days. Thus, tissue‐specific driver results likely provide the clearest estimate of the contribution of Acp29AB, indicating that its primary effect is to shift egg laying toward the earliest days after mating rather than to suppress total female fecundity. Although the magnitude of the observed effect was not large in absolute terms (a difference of less than 10 eggs across the first 3 days), it is large in relative terms (a 25% difference, which accounts for about a third of all female reproduction in our assays) and therefore could have important evolutionary implications.

From a female perspective, a shift to earlier reproductive timing may or may not carry fitness benefits. Because reproductive value typically declines with age, earlier reproduction should contribute disproportionately to lifetime fitness, especially under ecological conditions where mortality is high, resources are short‐lived, or populations are growing (Cole 1954; Williams 1957; Charlesworth 1970). Alternatively, early‐life reproduction could come at a substantial cost in terms of later‐life reproduction, for example via higher mortality that reduces lifetime reproductive success. Surprisingly little is known about survival of *D. melanogaster* in nature, although mark‐recapture experiments suggest high daily mortality and life expectancy under a week (Rosewell and Shorrocks 1987). We purposely designed our competitive fitness assay to emphasize early reproduction and female–female competition and detected no difference between females mated to control versus knockdown males. These results indicate that, at least under laboratory conditions, receipt of Acp29AB is effectively neutral for females. At the same time, it is easy to envision ecological contexts in which earlier reproduction would yield real benefits—for instance, if adult lifespan were short, or populations were growing exponentially. In such scenarios, the accelerated timing we document here could provide females with a fitness advantage. In an extreme world where only the earliest egg‐laying matters for fitness (e.g., very high adult mortality), females would likely benefit from receipt of Acp29AB.

Another potential pathway for costs or benefits to females is via indirect effects of SFPs on offspring quality. For example, Priest et al. (2008) found that female *D. melanogaster* with greater exposure to males died younger and had lower lifetime reproductive success, but daughters of these females showed higher lifetime reproductive success, suggesting costs paid by females for benefits reaped by offspring. Although our competitive fitness assay captures part of these potential indirect effects, by including competition between developing larvae, it does not assess the next generation's competitive ability as adults. These two concerns—the unknown timing of reproduction in natural fly populations and the potential indirect effects of SFPs on offspring—highlight a limitation of our study and similar ones. Fitness measures will be highly sensitive to the parameters of the assay, and subtle changes to the design might paint a different picture. One possible way to deal with this challenge is to use an experimental evolution approach and ask whether reduced expression of an individual SFP is favored or disfavored in different life history contexts, integrating across several generations of observed evolutionary change to estimate selection.

In summary, we identified a novel role for Acp29AB in promoting early oviposition, confirmed its contribution to male sperm defense, and showed that females experience no detectable fitness costs under our assays. Instead, the influence of Acp29AB on females appears selectively neutral or even potentially beneficial, depending on the ecological context. This pattern is consistent with the suggestion that rapid molecular evolution of some SFPs may occur without overt sexual conflict, potentially driven by fine‐scale interactions with sperm storage and fertilization rather than by persistent male–female arms races. These findings highlight the complexity of SFP effects and underscore the value of examining not only total reproductive output but also the timing of reproduction in understanding the evolution of interactions between males and females. Further work in both natural and laboratory contexts should reconstruct the *D. melanogaster* life history most relevant to SFP evolution, partition the fitness value of early‐life reproduction, and extend temporal analyses to other SFPs in order to clarify whether the evolutionary dynamics of SFPs are best understood through the lens of conflict, cooperation, or neutrality.

## Author Contributions

**Samantha Kincaid:** conceptualization (equal), data curation (lead), formal analysis (equal), funding acquisition (supporting), investigation (lead), methodology (equal), visualization (equal), writing – original draft (equal), writing – review and editing (equal). **Graham C. McLaughlin:** conceptualization (equal), formal analysis (supporting), funding acquisition (supporting), investigation (supporting), methodology (equal), writing – review and editing (supporting). **Sophia Sudak:** investigation (supporting), writing – review and editing (supporting). **Brian Hollis:** conceptualization (equal), data curation (supporting), formal analysis (equal), funding acquisition (lead), investigation (supporting), methodology (equal), project administration (lead), supervision (lead), visualization (equal), writing – original draft (equal), writing – review and editing (equal).

## Funding

This study was supported by funds from the U.S. National Science Foundation (award number 2212157) to B.H., a University of South Carolina SPARC grant to S.K., and a University of South Carolina Magellan grant to G.M.

## Conflicts of Interest

The authors declare no conflicts of interest.

## Supporting information


**Figure S1:** No difference in measured sperm offense (P2) between tissue‐specific Acp29AB knockdown males and control males (*n* = 33–39 per group), diamonds indicate mean.

## Data Availability

The datasets analyzed in this study are available on Dryad at https://doi.org/10.5061/dryad.c2fqz61pz.
