# Supplementary figures and images for: Seminal Fluid Protein Acp29AB Shifts Egg‐Laying Timing in Drosophila Without Detectable Effects on Female Fitness
Source: Ecol Evol. 2025 Dec 12;15(12):e72677. doi: 10.1002/ece3.72677 (PMC12700768; doi:10.1002/ece3.72677)

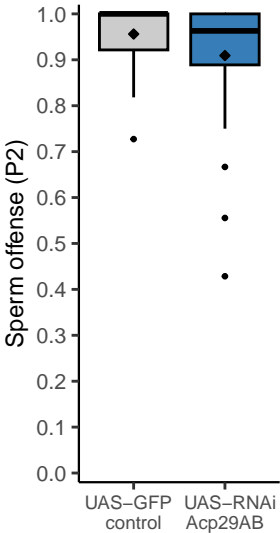

Supplement: Supplementary file 1 — Figure S1: No difference in measured sperm offense (P2) between tissue‐specific Acp29AB knockdown males and control males (n = 33–39 per group), diamonds indicate mean. [file ECE3-15-e72677-s001.pdf]
